# Oral cancer screening outcomes in the Latin American region with special relevance to Brazil and Cuba: a systematic review

**DOI:** 10.4317/medoral.26361

**Published:** 2024-02-18

**Authors:** Caique Mariano Pedroso, Ana Gabriela Normando, Maria Eduarda Pérez-de-Oliveira, Luciana Estevam Simonato, Mario Fernando de Goes, Ana Carolina Prado Ribeiro, Thais Bianca Brandão, Marcio Ajudarte Lopes, Saman Warnakulasuriya, Alan Roger Santos-Silva

**Affiliations:** 1 Oral Diagnosis Department, Piracicaba Dental School, University of Campinas (UNICAMP), Piracicaba, São Paulo, Brazil; 2 Dental School, University Brasil (UNIVBRASIL), Fernandoìpolis, São Paulo, Brazil; 3 Oral Medicine Service, Hospital Sirio-Libanes, São Paulo, Brazil; 4 ﻿Dental Oncology Service, Sao Paulo State Cancer Institute (ICESP-FMUSP), Sao Paulo, Brazil; 5 The WHO Collaborating Centre for Oral Cancer; 6 Faculty of Dentistry, Oral and Craniofacial Sciences, King's College London, London, UK

## Abstract

**Background:**

The Latin American region represents a hotspot for oral cancer incidence and mortality. To reduce oral cancer mortality rates, screening for early detection of subjects with suspicious or innocuous oral lesions has been promoted. A systematic review was performed to assess the outcomes of oral cancer screening in the Latin American region.

**Material and Methods:**

An electronic search was conducted in eight databases and grey literature. The eligibility criteria included screening where adult participants underwent any screening test during an organized screening program. Screening programs were assessed to understand trends in oral cancer diagnosis. Rates of oral cancers diagnosed in screening programs were classified as increase, decrease, or sTable based on each year assessed.

**Results:**

Following our searches, twelve studies conducted in Brazil and Cuba were included. The screening tests reported were visual oral examination (VOE) and in one study in addition light-based fluorescence testing. 13,277,608 individuals were screened and a total of 1,516 oral cancers were detected (0.01%). Only two studies aimed to screen high-risk individuals (smokers and drinkers). Oral cancer cases diagnosed during screening programs were proportionately sTable over the years 1997 to 2009 but increased from 2010 to 2021. The fluorescence-associated VOE test demonstrated a sensitivity of 100% and a specificity of 90%. Similarly, the VOE test alone exhibited a sensitivity of 100%, with specificity ranging from 75% to 90%.

**Conclusions:**

Screening studies conducted in Latin American countries had serious limitations both in methodology (lack of examiner training) and in reporting data (lack of description of clinical categories of screen positives). Capacitation of health workers to perform VOE in well-designed screening programs should be implemented.

** Key words:**Oral cancer, screening, early detection, diagnosis, systematic review.

## Introduction

Oral cancer is an important global health issue. An estimated 377,713 new cases and 177,757 deaths were reported by the Global Cancer Observatory (GLOBOCAN) in 2020 (1). Development of oral cavity cancer is known to be associated with lifestyles such as tobacco use (both smoking or smokeless forms) and alcohol consumption and for lip cancers ultraviolet exposure (2). Due to significant regional differences in the prevalence of well-recognized risk factors related to oral cancer, there is worldwide variation concerning disease incidence and mortality, with higher incidence found in lower- and middle-income countries, especially in Brazil and Cuba (1). In addition, in the Latin American and the Caribbean region, socioeconomic factors and economic inequalities may influence the incidence and mortality rate of oral cancer which is considered a socioeconomic-mediated disease (3).

Brazil represents the largest population in the Latin American region. In Brazil, as well as in other countries from Latin America, oral cancer poses a significant health burden (oral cancer is the 16th most common cancer in both sexes with an annual incidence of 3.6 cases per 100,000 inhabitants and a mortality rate of 1.5 deaths per 100,000 inhabitants) (1). This high oral cancer incidence makes it imperative to evaluate the effectiveness of cancer screening programs in this specific geographic environment and cultural context (1). While global data on oral cancer screening outcomes provide valuable insights (4,5), it is essential to recognize the impact of such programs may vary significantly depending on specific regional and cultural factors.

To reduce oral cancer mortality rates, screening for early detection of subjects with suspicious or innocuous oral lesions has been promoted. Screening programs contribute to advanced referral for performing diagnostic tests, biopsies, and histopathological analysis (4). Screening is defined as a method of identifying asymptomatic individuals who probably have a disease and applying tests, to distinguish them from those who may not have it (5). In this context, several models of oral cancer screening programs have been reported across the globe, including screening high-risk groups or screening the whole population, programs integrated with medical screening, industrial and workplace, mouth-self-examination (MSE), and opportunistic screening (5). The most common screening test executed is the visual oral examination (VOE), which involves an inspection and palpation of the oral cavity and lip under a suiTable light source (6). In addition to VOE, several adjunctive, non-invasive diagnostic tests are reported in the literature for oral cancer screening including light-based tests, vital staining, and oral cytology (7). Recently, salivary biomarkers and artificial intelligence have been proposed to detect oral cancer (8,9). Nevertheless, until now, all these adjunctive non-invasive methods offer limited evidence of effectiveness as they are still in stages of development and may be considered promising methods (5).

In this context, understanding the performance of screening programs undertaken for oral cancer screening is essential to refine future screening strategies, to reduce deaths related to the disease. The performance refers to the effectiveness and accuracy of an oral cancer screening program. It encompasses the ability of the screening program to achieve a high yield to correctly identify individuals with oral lesions or abnormalities (high sensitivity) and to correctly exclude individuals without oral lesions (high specificity). Therefore, the study aims to systematically review the performance and outcomes of all screening programs already performed in the Latin American region. The focused question for this review is: What are the performance and outcomes of screening tests and programs undertaken to detect oral cancer in adults in the Latin American region?

## Material and Methods

- Protocol and registration

This systematic review was conducted and reported according to the guidelines of Preferred Reporting Items for Systematic Reviews and Meta-Analysis of Diagnostic Test Accuracy Studies (PRISMA DTA-Statement) (10). The protocol was peer-reviewed and registered at the International Prospective Register of Systematic Reviews (PROSPERO) under the registration number: CRD42022329803.

- Eligibility criteria

The eligibility criteria were based on the following PIRD strategy (10) (Population, Index, Referential, and Diagnostic) to assess diagnostic accuracy tests: *P* - adult population; I - oral visual examination and/or other adjunctive non-invasive tests; R - surgical or biopsy with histological assessment; D - oral cancer.

We included cross-sectional test accuracy and descriptive studies that investigated the performance of screening to detect oral cancer in adult patients (18 aged or over) living in the Latin American region. Studies were considered if a screening test was applied to a selected group of individuals in an organized program and screen positives were then referred to a specialist to confirm a final diagnosis. We considered screening only asymptomatic individuals. In addition, only detection of oral squamous cell carcinoma located in the oral cavity and lip was included at following the sites: tongue, hard palate, buccal mucosa (lip and cheek lining), the floor of the mouth, retromolar trigone, and gingiva. Regarding noninvasive tests, for detection of oral cancer, we included: VOE, vital staining (toluidine blue), light-based detection (autofluorescence or chemiluminescence), remote screening (telemedicine), biomarkers (saliva and blood), oral cytology (brush biopsy), magnetic resonance imaging, or artificial intelligence.

We excluded: studies that did not specify the age groups; studies that included pediatric patients; studies that screened for cancer in anatomical sites other than the oral cavity and lip; studies reporting exclusively oral potentially malignant disorders; epidemiological surveys reporting disease prevalence; case finding reports, case reports, reviews, letters, short-communications, conference abstracts, and laboratory research; studies whose full texts were not available; studies conducted in other regions outside Latin America; studies published in a language other than English, Spanish, French or Portuguese; studies with duplicate data.

- Information sources and search strategy

We conducted literature searches in eight electronic databases: PubMed (via MEDLINE), Scopus, Embase, Web of Science, SciELO, LILACS, IBECS, and BBO-ODONTOLOGIA (last four databases were assessed via Virtual Health Library). Furthermore, additional searches were performed in the grey literature (Google Scholar, ProQuest Dissertation and Thesis, and ‘Biblioteca Digital de Teses e Dissertações’) and reference lists of included studies to find those missing in the search strategy. We conducted all searches on May 03rd, 2022, and we updated the search on August 10th, 2022.

We adapted the search for each database, including related medical subject headings (MeSH) and free terms controlled with boolean operators (OR and AND). We used the following keywords: "Adult" OR "Young Adult" OR "Middle Aged" OR "Aged" OR "Elderly" AND "oral cancer” OR "mouth neoplasm" OR "oral neoplasm” OR "mouth cancer" AND "screening" OR "early detection" OR "early diagnosis" OR "screening test” OR "sensitivity" OR "specificity" OR "reproducibility" OR "accuracy" OR "diagnostic accuracy". The search terms used in each database can be found in Supplement 1.

- Study selection

After searching databases and article selection, we performed the duplicate removal process in a two-step procedure. Firstly, one author (C.M.P.) exported records from each database to EndNote reference manager software (EndNote X7, Thomson Reuters, Philadelphia, PA), and the same author removed duplicate articles. Afterwards, the records were imported to the online software Rayyan (Rayyan, Qatar Computing Research Institute), and the same author manually removed residual duplicates in the second step. We applied a two-phase process to select the studies. In the first phase, two reviewers (C.M.P, M.E.P.O) selected articles based on titles and abstracts retrieved from databases using Rayyan. Studies that did not meet the inclusion criteria were excluded. Studies with titles and abstracts with insufficient information were directed to the next phase. In the second phase, the same reviewers applied the eligibility criteria to the studies' full text and recorded the reasons for exclusion. Two authors (C.M.P, M.E.P.O) first resolved any disagreement in both phases by discussing and then consulting a third author (A.G.N). One author (C.M.P) critically assessed reference lists for all eligible articles, and the same author selected the articles that were missed during our searches.

- Data collection process

Two independent reviewers (C.M.P, A.G.N) collected data from the selected articles and the retrieved information was cross-checked. Any disagreement was discussed between them and the third reviewer (S.W) if necessary. The following data were extracted: first author; year of publication; country; characteristics of the participants (number, age, sex, and risk factors); type of screening test, number of examiners and whether calibration was performed, number of oral cancers detected, study analytics (true-positive, true-negative, false-positive, false-negative), methods of recruitment of participants, and additional strategies reported in screening programs.

- Risk of bias and applicability

We used the Quality Assessment of Diagnostic Accuracy Studies 2 (QUADAS-2) tool to assess the quality of the included studies that assessed the screening tests over four key domains: patient selection, index test, reference standard, and flow and timing of participants through the study (11). Each domain was evaluated in terms of risk of bias and applicability. The included studies were rated as high, unclear, or low according to the qualification domains. A third reviewer (A.G.N) was consulted in case of disagreements. All reviewers agreed on the scoring before critical appraisal assessments. Furthermore, we used the Joanna Briggs Institute (JBI) Critical Appraisal Checklist for Analytical Cross-sectional Studies (12), which evaluated the performance of screening programs. We categorized these studies as follows: high risk of bias (when the study reached a score of up to 49% "low"), moderate risk of bias (when the study reached a score of 50% to 69% "low"), and low risk of bias (when the study reached at least a score of 70% "low").

- Effect measures and synthesis of results

The primary objective of this study was to evaluate the performance of screening tests and the outcomes of programs designed to detect oral cavity cancers in Latin America. We examined whether reported studies provided sufficient data to determine the sensitivity and specificity estimates, positive predictive value (PPV), and negative predictive value (NPV). Due to the absence of crucial data in the included studies, a meta-analysis of diagnostic accuracy could not be performed. The non-reporting of the number of suspicious cancer lesions detected by tests in several studies precluded the conduct of standard analyses such as the Diagnostic Odds Ratio (DOR) and Summary Receiver Operating Characteristic Curve (SROC). The quantitative data, including the number of individuals screened and oral cancer cases diagnosed, were compiled to create a database for analysis. The relative frequency was used to report the overall estimates of oral cancer. We summarized the yearly data of individuals screened and oral cancer cases diagnosed to determine whether there was a rise, fall, or consistency of cases diagnosed over time. A line chart was created based on the extracted data.

## Results

- Study Selection

**Figure 1:**
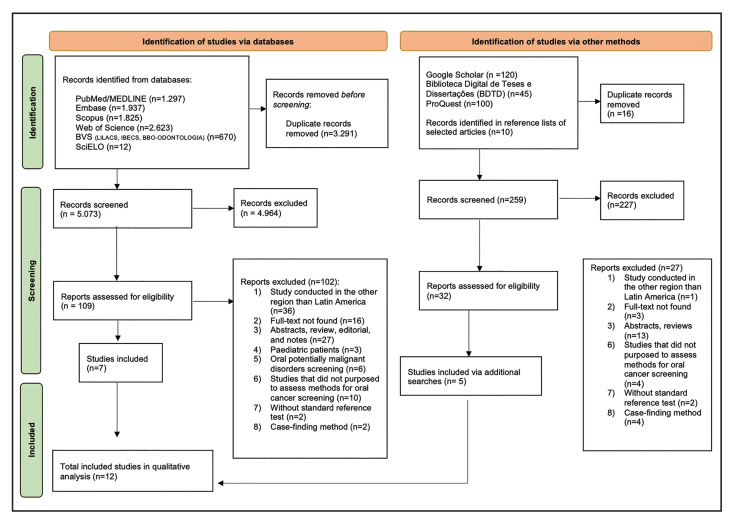
Flowchart describing literature search and overall included studies according to PRISMA guideline (2020).

After a systematic search, we identified 8,364 records in the primary electronic databases. After duplicate removal, 5,073 records were screened by their title and abstract. In phase one, we considered 109 studies for full-text reading. After evaluation by the eligibility criteria, 102 reports were excluded in phase two, and we included 07 studies. In additional searches, we identified 275 records in the grey literature and reference lists of included articles. After duplicate removal, we screened and evaluated 259 records. Subsequently, we assessed 32 reports for eligibility criteria. After full-text reading, 27 reports were excluded, and we included 05 studies. Overall, we included 12 studies (Supplement 2) in our systematic review (Fig. 1).

- Study characteristics

**Figure 2:**
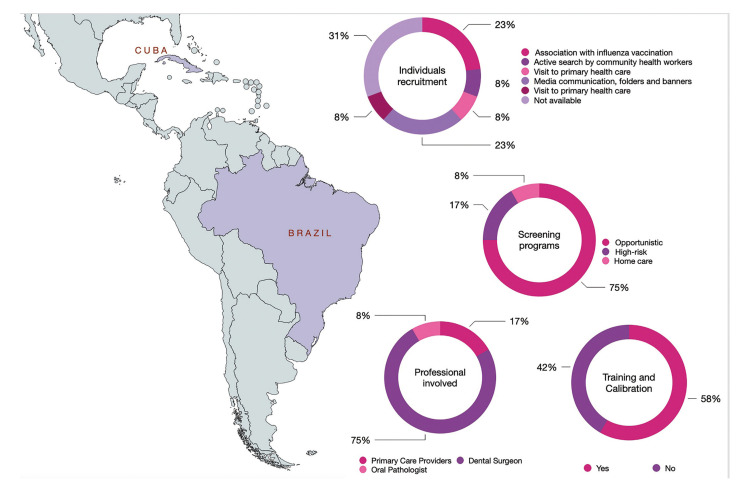
Characteristics of screening programs in Brazil and Cuba concerning individual recruitment, type of screening, professionals involved, and training and calibration of screening tests.

The period of publication of all included studies ranged from 1997 to 2021. These 12 studies were conducted in two countries located in the Latin American region: Brazil (*n*=11) and Cuba (*n*=1). Only two screening tests were reported: VOE and light-based tests (fluorescence). The population screened in each study ranged from 143 to 10,167,999 participants (total *n*=13,277,608) (Table 1), and a total of 1,516 oral cancers were detected. Regarding gender with available data, females (*n*=4,085) were screened more frequently compared to males (*n*=3,490). The screening was performed in opportunistic settings (75%) and among high-risk individuals (17%) (Fig. 2). Opportunistic screening takes place when a dental health professional provides an additional examination or test to a patient during a routine dental check-up. High-risk screening specifically aims to detect lesions in patients who smoke or consume alcohol. Dental surgeons (75%), primary care providers (17%), and an oral pathologist (8%) were the professionals responsible to execute the screening (Fig. 2). Only eight studies (58%) reported training and calibration of these professionals to perform screening (Fig. 2). Only 2 studies provided sufficient data to analyze the accuracy of screening (Table 1).

- Risk of bias and applicability

In diagnostic accuracy studies (*n*=4), all studies have a clear description (low risk of bias) of the patient selection method, one study (25%) had gaps for describing the execution of index and reference standard tests, and 75% (high risk of bias) of studies did not report the interval between application of the index text and re-examination (Supplement 3). For applicability concerns, the included studies were matched with the review questions, especially reference standard and patient selection, of which 75% and 100% were low proportions, respectively (Supplement 3).

For descriptive studies (*n*=8) that assessed the screening, one study was classified as low risk, five as moderate risk, and two as high risk of bias (Supplement 3). The majority of descriptive studies did not describe the eligibility criteria to include the individuals. Also, five studies did not report clearly how VOE was performed and whether the examiners were calibrated prior to the screening process.

**Table 1:**
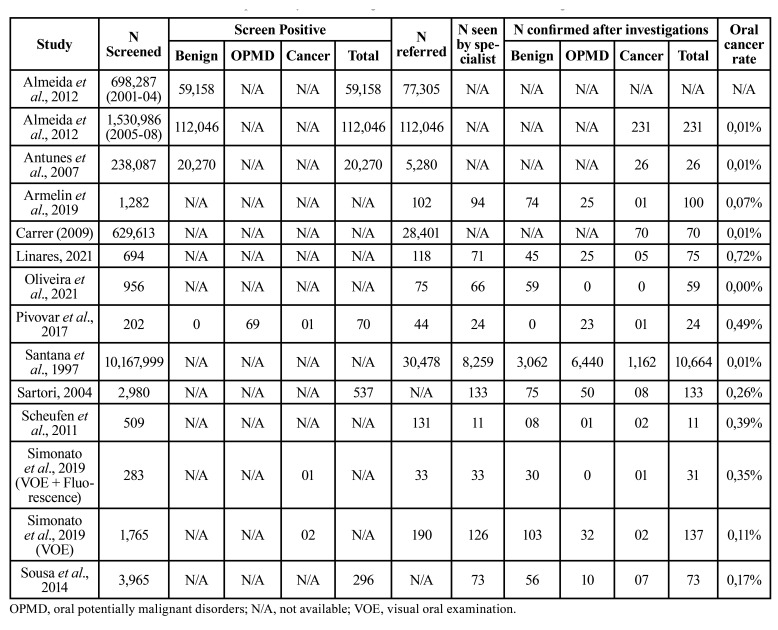
Overview of individuals screened, positive by test, and diagnosed with oral cancer in screening studies.

- Results of individuals studies

Brazilian studies: In Brazil, most screening programs were targeted towards older populations (>60 years) and were conducted as part of an organized influenza vaccination program during the period from 2003 to 2009. Screening was offered to high-risk individuals, namely smokers and alcohol drinkers, in two studies. In all studies the screening tests involved the use of visual oral examination (VOE) and in one study additional fluorescence tests were conducted. Lip and oral cavity screenings were conducted by dental surgeons, oral pathologists, and primary care providers. Additional strategies included leaflet distribution and instructions for MSE to aid oral cancer prevention and to improve the awareness of the population on the risk habits and early symptoms.

Overall, 3,109,609 individuals were screened, and the proportion of oral cancers detected corresponds to 0.01% of the screened population. Oral cancer detection rate ranged from 0.01% to 0.72% in individual studies (Table 1). Only two studies reported the number of screen positives by recording the number of subjects with suspected oral cancers. Most studies reported only the total number of positive subjects without separately recording those with benign lesions, OPMD, or cancer (Table 1). We therefore could not undertake any detailed analysis on the efficacy of screening, except for 2 reported studies.

Cuban study: A screening study conducted in Cuba from 1983 to 1990 was included in this analysis, which was performed in an opportunistic setting. The screening involved VOE performed by dental surgeons on 10,167,999 individuals, out of which 8,259 were assessed by specialists to confirm the diagnosis. The study did not report the number of suspicious cancers found by VOE. The final diagnosis of malignancies constituted 0.1% of the screened population. Most of the diagnosed oral cancers were in stages III from 1982 to 1984, and stages I and II from 1985 to 1988.

- Synthesis of results

The VOE and fluorescence sensitivity/specificity values were available only in two studies (Table 2). VOE reported 100% for sensitivity and specificity ranged from 75% to 90%. The light-based fluorescence had 100% and 90% for sensitivity and specificity, respectively (Table 2). PPV for VOE ranged from 0.36 to 0.43 and VOE associated with fluorescence showed a PPV of 0.37%. NPV was 0.10 for both screening tests (Table 2).

**Figure 3:**
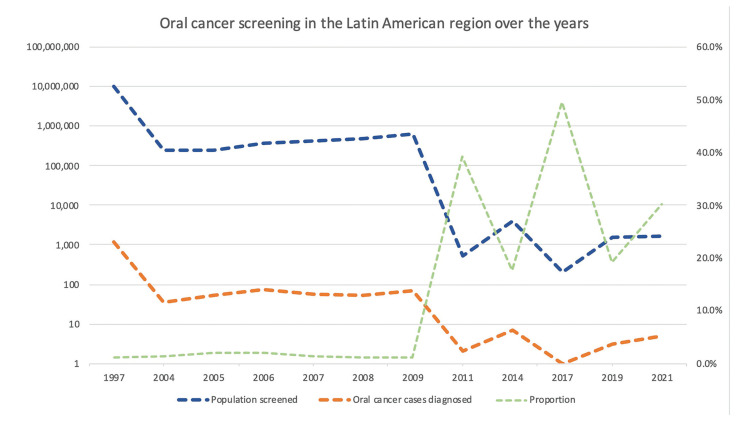
Oral cancer screening over the years in Latin America represents the number of individuals screened and cases detected each year reported.

During the screening period (1997 to 2021), it is possible to observe a decrease in the number of individuals screened and a consequent reduction in oral cancer diagnosis (Fig. 3).

**Table 2:**
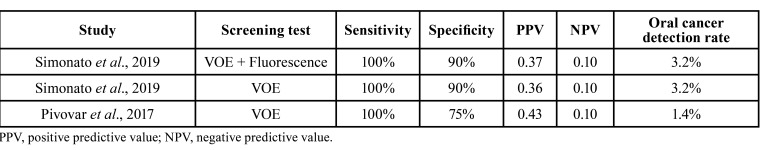
Diagnostic values of screening tests.

Since the 2000s, the number of diagnosed oral cancer cases has decreased significantly. There was relative stability in the number of cases of oral cancer from 2004 to 2009, since the variation in the number of cases in this period was relatively small. The data also showed that in some specific years, such as 2005 and 2006, the number of oral cancer cases diagnosed increased significantly compared to previous years. However, the number of diagnosed oral cancer cases gradually increased again, reaching 71 cases in a population of 629,613 people in 2009. When analyzing the data for the screened population, the oral cancer rate per person screened remained relatively sTable from 1997 to 2009, with small variations over the years. However, between 2009 and 2021, there was a significant increase in the oral cancer rate per person screened, with an increase in cases diagnosed relative to the population screened (Fig. 3).

## Discussion

VOE is considered a conventional test to identify individuals with lesions suspicious of oral cancer. The sensitivity and specificity estimates reported in two of the included studies for VOE are within the ranges reported in a previous global analysis (13). However, both sensitivity estimates in our study are from small sample size studies, which makes it difficult to affirm the effectiveness of the diagnostic test. In comparison to the previous global analysis that assessed VOE to detect oral cancer lesions, the study's sensitivity estimates are generally lower. This could be due to several factors, such as a smaller sample size, differences in the methodology used, or the inclusion of patients with more advanced or difficult-to-detect oral cancer. It is also possible that the examiners had less experience or training in conducting VOE, affecting the accuracy of their assessments. On the other hand, the study's specificity estimates are within the range reported in previous study (13), which suggests that VOE may be relatively effective in ruling out individuals who do not have oral cancer. This is an important consideration in oral cancer screening, as false positives can lead to unnecessary diagnostic tests and procedures. Overall, the variability in sensitivity and specificity estimates across studies highlights the importance of careful methodology and standardization in conducting oral cancer screening using VOE.

The screening study reported by Simonato *et al*. (14) is the first instance when an autofluorescence adjunctive technique has been applied in a primary care setting. All previous studies to our knowledge have been conducted in secondary care facilities (15). The sensitivity estimates for fluorescence testing reported in this study are higher than the sensitivity estimates reported in the global analysis (15). This suggests that fluorescence testing could be a reliable tool for detecting oral cancer in Brazil. However, a single study with a small sample size has limitations, and reproducibility studies are needed in different patient populations. Previous evidence reported that the fluorescence test has limitations due to poor specificity and this test has not been adequately assessed in the primary care setting (16). It may also be useful to explore complementary screening methods in future screening studies, such as combining toluidine blue staining and fluorescence testing with VOE, to improve the accuracy and reliability of oral cancer detection.

The results of the two studies of the VOE test showed a range of PPV for oral cancer screening exams, with values ranging from 0.36 to 0.43. These results suggest that the accuracy of oral cancer screening exams may vary depending on the specific population being screened and the method used. One potential factor that could contribute to the variation in PPV values is the prevalence of oral cancer in the population being screened. If the population being screened has a higher prevalence of oral cancer, this could lead to higher PPV values, as the screening exam is more likely to correctly identify individuals with the disease. Conversely, if the population has a lower prevalence of oral cancer, this could lead to lower PPV values, as the screening exam is more likely to produce false positive results. However, both included studies in PPV analysis comprises a small simple size. The small simple size in these two screening programs may affect the precision and generalizability of the PPV estimates and may not be as meaningful or reliable for drawing robust conclusions. While the small sample size restricts the PPV's interpretability, there is a necessity of conducting larger-scale studies to obtain more accurate and reliable estimates of the PPV for future oral cancer screening programs. Overall, the results of these studies suggest that the accuracy of oral cancer screening examinations may vary depending on the specific population being screened and the method used. Clinicians should consider these factors when interpreting the results of screening exams and making decisions about further diagnostic testing or treatment. Further research is needed to identify the most accurate and effective screening methods for oral cancer, particularly in populations with varying levels of disease prevalence.

During the period of the screening, there was a greater period of stability (2004-2009) of diagnosed cases proportional to the number of individuals who participated in the screening programs. The number of oral cancer cases detected during a screening program may vary depending on several factors, such as the health literacy of the target population, the acceptability of the screening methods for a given population, and the effectiveness of the program in identifying early-stage cancer cases (15-17). Oral cancer may be more likely to be diagnosed by dental surgeons who are adept at recognizing the clinical signs of the disease (5). In this context, as part of screening programs and public health campaigns, VOE was found to be valid for the detection of oral cancer in the previous study but it still suffers low consistency of application and the need for calibration of the examiners (17). In addition, VOE has been considered a cost-effective strategy for oral cancer detection, especially in an opportunistic and high-risk patient setting (18). However, this non-invasive method depends on the abilities of examiners and their knowledge about the clinical features of the disease at the time of screening, to improve the test’s sensitivity. Therefore, more training and calibration of examiners involved in oral cancer screening should be performed to have a better achievement of lesion detection by VOE.

We observed that several studies did not report whether the examiners were calibrated or trained to perform screening through VOE, which can explain the risk of bias as low certainty of evidence in included studies. Regarding the examiners, general dentists, oral pathologists, and primary care providers (PCPs) were cited as the health professionals utilized in these studies. For better performance of screening tests, general dentists are considered the best health professionals with sufficient knowledge to execute the tasks and improve test sensitivity (19). Nevertheless, as the Latin American region is considered low-and-middle-income countries, the training of general dentists can be compromised due to difficult access to higher education resulting in a low manpower ratio of these professionals (4,5). A previous study reported that otolaryngologists could perform screening with satisfactory sensitivity and specificity rates through VOE (20). In this context, to compensate for the lack of general dentists, PCPs and medical workers are suiTable health professionals able to perform oral cancer screening, as was reported by two included studies (21-24). Future national screening programs should select PCPs and medical workers and train them on how to recognize oral lesions facilitating geographic regions such as Latin America where there are restrictions on general dentists. Also, continuing education for dentists needs to be freely available to improve VOE sensitivity. The lack of training of examiners and not adhering to a standard method of application could explain the non-accepTable performance of the reported studies. Where there is a lack of trained oral health professionals working in primary care, MSE could be recommended to the population as a means of screening (25). The overall impression gained from this systematic review is that it is important for examiners and Principal Investigators to improve their skills in reporting test results following screening programs. In this regard an intervention campaign conducted in Argentina that included training dentists in diagnostic skills and a public awareness program through media and networks resulted in improving delays of diagnosis of oral cancer (26) and a reduction in mortality rates 10 years after a continuous campaign.

High-risk population screening is a model for the early detection of oral cancer. Two included study from Brazil that performed VOE in a high-risk population suggests the possibility of applying a high-risk-oriented approach. Also, a clinical trial from India reported a reduction of 34% in oral cancer in high-risk populations after screening (27). From this perspective, targeted screening programs may be an effective strategy for reducing oral cancer mortality. Despite a limited number of published studies so far reported recruiting high-risk individuals (smokers and alcohol drinkers), this type of screening program could achieve good results (28). Therefore, identifying and directing these risk-high individuals towards appropriate preventive measures could be an alternative plan of action to implement in the Latin American region as primary oral cancer prevention.

Concerning screening programs undertaken in Latin American countries, there is only one opportunistic screening model implemented in Cuba through VOE (29), and the results suggest over several years of screening a reduction in advanced oral cancer cases; however, the Cuban screening program did not eventually lead to a reduction in the incidence and mortality rate (29). Not only in the Latin American and Caribbean region but also in Europe, where some countries are considered a hotspot for oral cancer incidence, do not report consistent results regarding established programs for oral cancer detection (30). Taiwan has demonstrated success with VOE use in its screening program, resulting in reduced oral cancer incidence and mortality rates (31). Most of the included studies in this systematic review are from Brazil, where high oral cancer mortality rates are reported in the literature (32,33). The lack of well-researched programs denotes that further clinical trials are necessary to standardize oral cancer screening programs, especially in Latin American countries, to improve the efficacy of VOE which will eventually lead to a reduction in mortality rates.

 The findings in our systematic review are subject to a number of limitations. Our searches brought up only a few studies in two countries in Latin America, which restricts our findings to these regions. Many studies did not report the effect measure, which restricted our analysis. Despite studies aimed to report their performance of screening, it was possible to observe the absence of crucial data, such as the absolute number of oral cancer-positive cases detected by testing, which compromised the standard analysis. It was not possible to undertake a meta-analysis. Primary studies remained with several limitations in methodology. In this context, further clinical trials are needed to derive more reliable information regarding the role of VOE as a screening test, calibration of the screeners, high number of screened populations, and necessary follow-up process to confirm false positives, to assess the reduction in mortality from oral cancer as an outcome of screening programs.

In summary, only a limited number of screening programs are reported for oral cancer detection in Latin American countries. The precise results from VOE and light-based (fluorescence) screening tests used by general dentists, oral pathologists, and primary care providers to perform oral cancer screening were not easily accessible for analysis due to poor reporting in the published reports. The results of oral cancer detection during the screening remained sTable over the years elucidating the necessity of better improvement, especially concerning target population and VOE applicability.

## Conclusions

The results of visual oral examination (VOE) yielded several limitations in the performance of oral cancer screening in Brazil mainly due to a lack of examiner training and poor data reporting. Despite the reduction in the number of cases of oral cancer diagnosed in the 2000s, the disease still represents a public health problem and requires continuous attention. National screening programs for oral cancer detection are still unavailable in Latin America and Caribbean countries. Thus, national screening programs should be further tested to achieve early detection, and any reduction of oral cancer incidence and mortality rates.

## Data Availability

Data supporting the findings of this study is available on the supplementary material and from the Correspondence upon reasonable request.
